# Microbial oil-degradation under mild hydrostatic pressure (10 MPa): which pathways are impacted in piezosensitive hydrocarbonoclastic bacteria?

**DOI:** 10.1038/srep23526

**Published:** 2016-03-29

**Authors:** Alberto Scoma, Marta Barbato, Emma Hernandez-Sanabria, Francesca Mapelli, Daniele Daffonchio, Sara Borin, Nico Boon

**Affiliations:** 1 Laboratory of Microbial Ecology and Technology (LabMET), University of Gent, Coupure Links 653, B 9000 Gent, Belgium; 2 King Abdullah University of Science and Technology (KAUST), Biological and Environmental Sciences and Engineering Division, Thuwal 23955-6900, Kingdom of Saudi Arabia; 3 Department of Food, Environmental and Nutritional Sciences (DeFENS), University of Milano, Via Celoria 2, 20133, Milano, Italy

## Abstract

Oil spills represent an overwhelming carbon input to the marine environment that immediately impacts the sea surface ecosystem. Microbial communities degrading the oil fraction that eventually sinks to the seafloor must also deal with hydrostatic pressure, which linearly increases with depth. Piezosensitive hydrocarbonoclastic bacteria are ideal candidates to elucidate impaired pathways following oil spills at low depth. In the present paper, we tested two strains of the ubiquitous *Alcanivorax* genus, namely *A. jadensis* KS_339 and *A. dieselolei* KS_293, which is known to rapidly grow after oil spills. Strains were subjected to atmospheric and mild pressure (0.1, 5 and 10 MPa, corresponding to a depth of 0, 500 and 1000 m, respectively) providing n-dodecane as sole carbon source. Pressures equal to 5 and 10 MPa significantly lowered growth yields of both strains. However, in strain KS_293 grown at 10 MPa CO_2_ production per cell was not affected, cell integrity was preserved and PO_4_^3−^ uptake increased. Analysis of its transcriptome revealed that 95% of its genes were downregulated. Increased transcription involved protein synthesis, energy generation and respiration pathways. Interplay between these factors may play a key role in shaping the structure of microbial communities developed after oil spills at low depth and limit their bioremediation potential.

Mineralization of hydrocarbons to non-toxic compounds in marine environments is a process known to occur both in natural and anthropogenically-affected ecosystems^1^. Despite the fact that hydrocarbons are naturally released into the environment through bio- and geological processes^2^ oil spills have a major environmental impact. Temperature, pH, nutrient and O_2_ availability are known to affect the structure of seawater microbial communities^3^ along with the alkane profile of the spilled oil, thereby determining the long-term impact of oil spills. Another critical factor for oil biodegradation at sea is hydrostatic pressure. Pressure increases about 0.1 MPa every ten meters of seawater, meaning that for instance marine life at 1000 m below the water surface experiences a pressure equal to 10 MPa. Oil spills on surface waters are characterized by the release of hydrocarbons in the form of a thin film, mainly constituted by light, water-soluble hydrocarbons^4^. Eventually, these compounds dissolve in the water column or remain trapped within immiscible oil components, which become emulsified and are dispersed as small droplets^5^. Oil presence on the seafloor may be due to sinking, which is influenced by natural and anthropogenic factors as association with particulate^6^ and use of dispersants during oil spill control operation^7^, or may result from drilling activities, operation or accidents occurring at deep sea. In the recent Deep Water Horizon spill occurred in the Gulf of Mexico the fraction eventually sinking to the seafloor was estimated to be up to 10% of the total spilled oil, covering an area estimated between 3000–8000 km^2 ^8,9.

Information on microbial oil degradation mechanisms under hydrostatic pressures is scarce^10^,^11^,^12^,^13^ and focuses quite exclusively on high hydrostatic pressure (from 15 to 50 MPa, correspondent to depths >1500 m). Our hypothesis was that a mild increase in pressure (from 0.1 to 10 MPa) might be sufficient to impose significant stress on cells adapted to atmospheric pressure. Pressure increase in a water body follows the linear equation for vertical pressure variation (Equation 1)


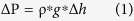


where ΔP represents the variation in pressure, *ρ* is the density of seawater, *g* is gravity and Δh is the difference in depth. Marine life in the mesopelagic zone (200 to 1000 m) is subjected to mild hydrostatic pressures (2 to 10 MPa). However, the relative pressure increase experienced in this zone is much higher with respect to deeper zones, as pressure doubles 5 times within 800 m. Other critical factors such as pH and salinity are not as remarkably affected by depth as pressure, while temperature varies significantly in this zone although to a lower extent with respect to pressure and depending on latitude and season^14^.

The study of how microbes residing in the surface of the seawater column respond to mild pressure increases may provide insights on the primary drivers for microbial diversity and their potential function in marine areas under anthropogenically-affected conditions. In the present paper, we focused on the effects exerted by mild pressure increases (0.1, 5 and 10 MPa, corresponding to 0, 500 and 1000 m depth) on two strains belonging to different species of the genus *Alcanivorax*, namely *A. jadensis* KS_339 and *A. dieselolei* KS_293, which were isolated from surface waters of the Mediterranean Sea. As these species are ubiquitous hydrocarbonoclastic marine bacteria detected in a wide range of oil-contaminated environments, from surface to deep sea^15^, in both seawater and sediments^16^,^17^,^18^, their response to pressure may provide interesting information on actual site environmental constraints following oil spills. Both strains showed a piezosensitive profile, with reduced growth yields under hydrostatic pressures higher than the atmospheric. Cell integrity and activity in *A. dieselolei* KS_293 was nevertheless preserved despite pressure increases up to 10 MPa and therefore was selected for comparing transcript pathways during growth at 0.1 and 10 MPa.

## Results

### Growth yields of Alcanivorax species under atmospheric and mild hydrostatic pressure

**Figure 1:**
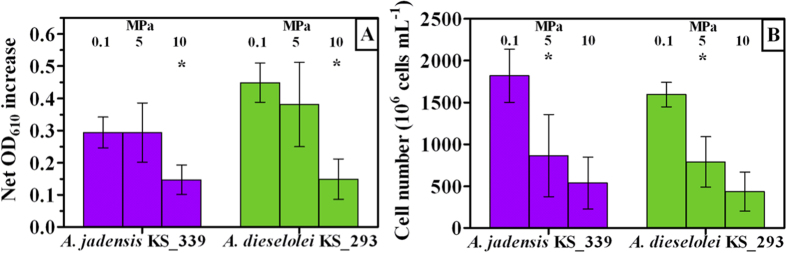
Growth of *A. jadensis* KS_339 (purple) and *A. dieselolei* KS_293 (green) under atmospheric (0.1 MPa) and mild pressure (5 and 10 MPa). Bars indicate 95% confidence intervals. A: Net optical density increase; asterisks indicate that values at 10 MPa were significantly lower (P < 0.05) than those observed at 0.1 and 5 MPa. B: Final cell number; asterisks indicate that values at 0.1 MPa were significantly higher (P < 0.05) than those observed at 5 and 10 MPa.

*Alcanivorax jadensis* KS_339 and *A. dieselolei* KS_293 were independently incubated at different hydrostatic pressures (0.1, 5 or 10 MPa) providing C12 as sole carbon source. Pressure had a major impact on cell growth, as estimated by optical density (Fig. 1a). At atmospheric pressure (0.1 MPa) *A. jadensis* had a lower cell density in comparison with *A. dieselolei*. However, when cells were incubated under the highest pressure (10 MPa) net growth was comparable between the two strains and lower than that observed at 0.1 MPa (*P* < 0.05) (Fig. 1a). Growth trends estimated by OD were different when cell number was analysed by flow cytometry, as strains were comparable at any given pressure (Fig. 1b). Increases in pressure up to 5 MPa resulted in a significant reduction of cell number (*P* < 0.05), which was further reduced upon incubation at 10 MPa. Reduction in growth between 0.1 and 10 MPa in both *A. jadensis* and *A. dieselolei* was equal to one order of magnitude. Provided that increased hydrostatic pressure would have resulted into a higher O_2_ solubility, control experiments under ambient pressure were carried out using pure O_2_ as gas phase (Fig. S2). As no effect on culture growth was detected, the potentially negative impact of increased oxygenation on cell replication was excluded.

**Figure 2:**
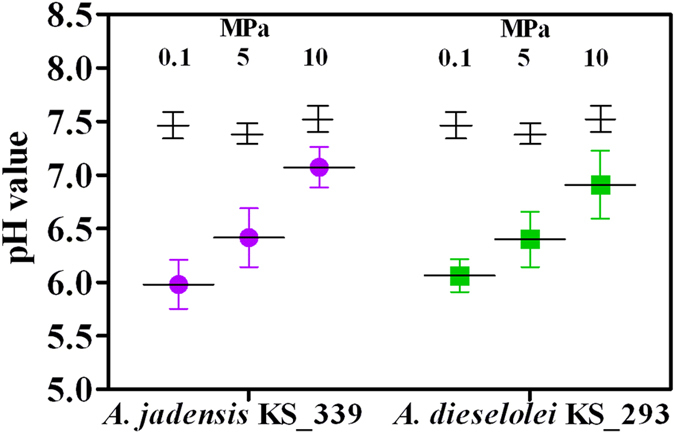
Decrease in pH value after incubation of *A. jadensis* KS_339 (purple) and *A. dieselolei* KS_293 (green) under atmospheric (0.1 MPa) and mild pressure (5 and 10 MPa). Bars indicate 95% confidence intervals. Black crosses represent sterile controls.

**Figure 3:**
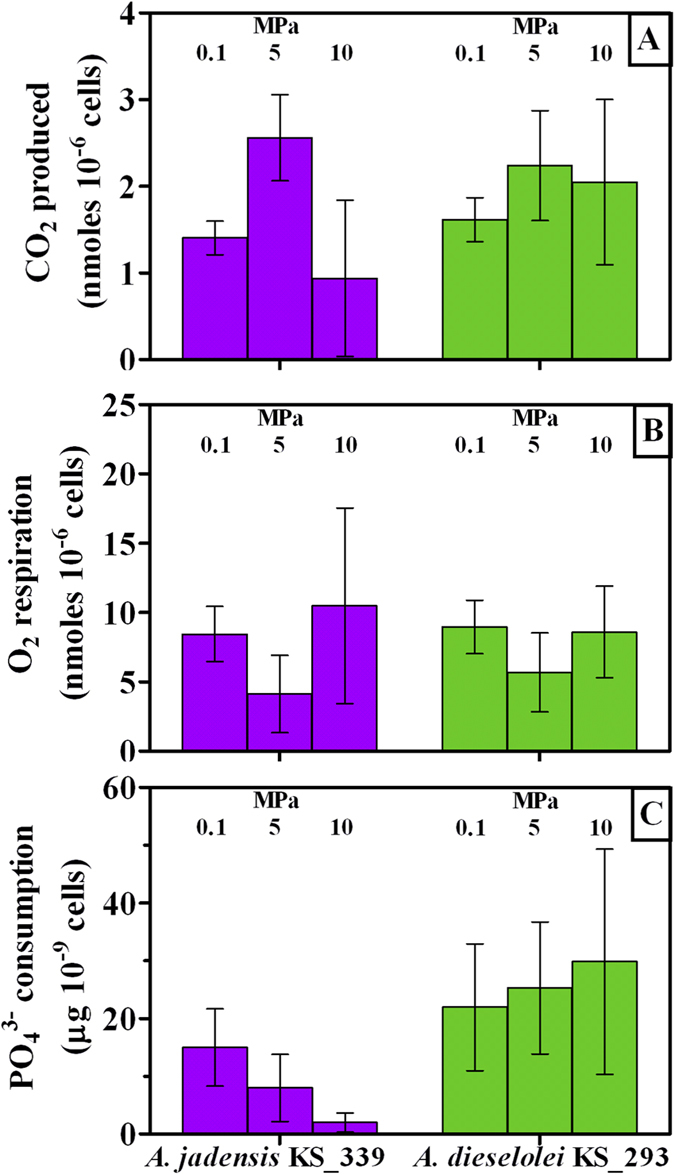
Physiological response in *A. jadensis* KS_339 (purple) and *A. dieselolei* KS_293 (green) under atmospheric (0.1 MPa) and mild pressure (5 and 10 MPa). Bars indicate 95% confidence intervals. All values refer to the final cell number and amounts detected at the end of the incubation. (**A**): CO_2_ production per cell; (**B**): O_2_ respiration per cell; (**C**): PO_4_^3−^ uptake per cell.

**Figure 4:**
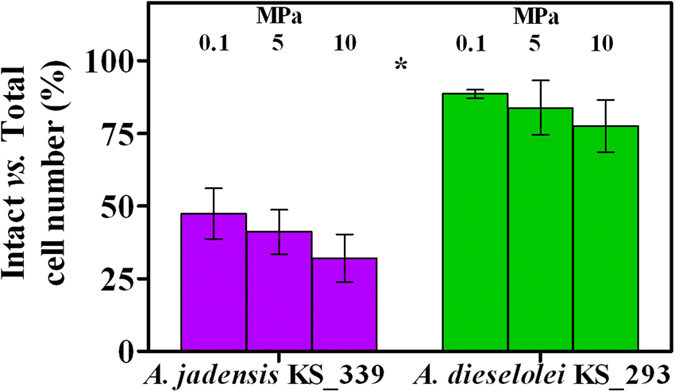
Cell integrity in *A. jadensis* KS_339 (purple) and *A. dieselolei* KS_293 (green) under atmospheric (0.1 MPa) and mild pressure (5 and 10 MPa) expressed as percentage of intact cells over total number of cells. Bars indicate 95% confidence intervals. Asterisk indicates that *A. dieselolei* KS_293 cells were always significantly higher (P < 0.05) than *A. jadensis* KS_339 cells at any tested pressure.

C12 water apparent solubility was determined (Fig. S1) and concentrations changed according to culture conditions and resulting cell yields, being 7.5 g L^−1^ the amount initially supplied (*i.e*, 1%, v-v). Detectable C12 in cell suspensions is affected by several phenomena (adsorption to plastic components of the reactor, oil solubility in water, apparent solubilisation by extracellular and transmembrane surfactants, residual C12 concentrations after cell consumption)^19^. Therefore, analysis of the detectable C12 was only aimed at evaluating its general bioavailability. Lowest C12 concentrations coincided with high cell number (32 and 98 μg L^−1^ in *A. jadensis* and *A. dieselolei*, respectively, at 0.1 MPa, Fig. S1 and Fig. 1b), indicating that access to the carbon source was not limiting. Provided that C12 was the sole carbon source supplied, decreased pH value as a result of CO_2_ production was considered an indirect measure of C12 degradation. Hence, a final low pH (Fig. 2) was considered as an indication of a high C12 consumption rate. Increased hydrostatic pressure was coincident with a minor change in pH in comparison with control experiments provided with sterile medium (Fig. 2), in agreement with reduced growth rates (Fig. 1b). The reduced amount of CO_2_ produced per reactor (Fig. S3) is consistent with such a pH increasing trend with pressure (Fig. 2), although it can be assumed that under increased hydrostatic pressure cells actually experienced more acidic pH values than what measured after decompression. In *A. jadensis* cell activity was increased at 5 MPa but eventually dropped to low values when tested at 10 MPa (CO_2_ production per cell, Fig. 3a), while *A. dieselolei* cells showed a constant output irrespective of the pressure applied (Fig. 3a). Thus, in the latter reduced impact on pH variation under pressure was a result of a lower cell number per volume rather than a decrease of the cell respiration rate. Concerning O_2_ respiration, no significant difference was detected in both strains, although reproducibility was higher when testing *A. dieselolei* (Fig. 3b). The final amount of O_2_ transferred to the liquid phase as a result of the higher hydrostatic pressure was also measured (Table S1) and was found to be of the same order of magnitude of cell respiration (Table S2). Strain activity was evaluated by the uptake rate of PO_4_^3−^ (Fig. 3c) whose consumption per cell decreased linearly with pressure in *A. jadensis* (Fig. 3c), while in *A. dieselolei* it remained constant (around 25 mg 10^−9^ cells) and higher than in *A. jadensis* (*P* < 0.05). The observed differences between the two strains are in accordance with the intact *vs* total cell ratio, which was higher for *A. dieselolei* at any given pressure (*P* < 0.05, Fig. 4). In *A. dieselolei* the amount of damaged cells at the highest pressure applied was rather low (about 22% of total cell number, Fig. 4) suggesting that despite the decreased growth rate in this strain some structural or physiological mechanism linked to cell integrity counterbalanced mild hydrostatic pressure.

### Transcriptomic response in A. dieselolei KS_293 at 1 and 10 MPa

**Figure 5:**
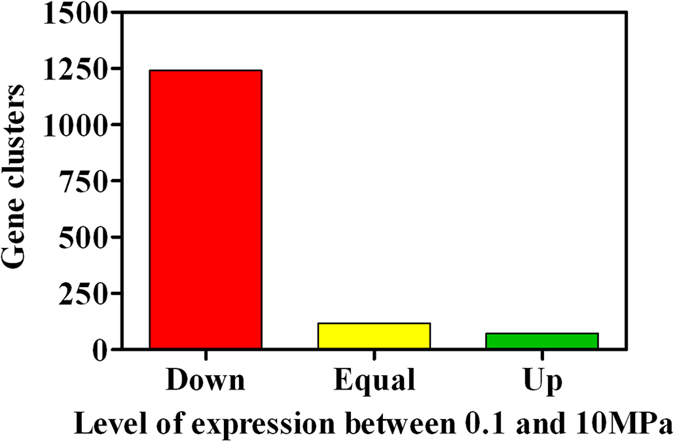
Effect of 4-day incubation at 10 MPa on the gene expression of *A. dieselolei* KS_293 cells with respect to 0.1 MPa.

**Figure 6:**
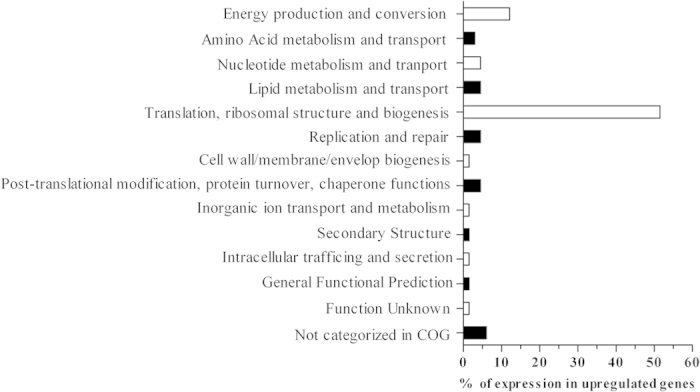
Upregulated COG in *A. dieselolei* KS_293 cells incubated at 10 MPa with respect to expression levels at 0.1 MPa. Percentage of expression is normalized on the total number of upregulated gene clusters, as shown in Fig. 5, green bar.

To assess the mechanisms determining the observed response to hydrostatic pressure, *A. dieselolei* KS_293 cells grown under 0.1 and 10 MPa where collected and their transcriptome analyzed. Under 10 MPa the majority of the genes were downregulated as compared to atmospheric pressure (Fig. 5). Gene clusters showing upregulation between 0.1 and 10 MPa (here defined as a log2 fold change higher than 0.50) were only 70 out of 1430 (equal to 4.9% of all SSD gene clusters). This result alone gives a hint of the general impediment imposed over cell metabolism by a pressure of 10 MPa, suggesting that critical metabolic pathways for cell replication and survival were impaired. Clusters of orthologous genes (COG) upregulated under pressure were predominantly associated with translation, ribosomal structure and biogenesis, and energy production and conversion (Fig. 6).

The most represented class of upregulated genes was that associated with ribosomal proteins (28/66, Table 1), in particular genes related with the 50 S subunit (21/28). On the contrary, all RNA ribosomal methyltransferase proteins were downregulated (10/10, Table 1). Genes related with tRNA modification were also upregulated, in particular *truB*, a tRNA pseudouridine synthase, which was the third most upregulated gene in the whole transcriptome (3.09 log2 FC, Table 2). Translation-related elongation factors were largely upregulated or unaffected (5/6); in particular, one Tu and Ts were upregulated (Table 2). Similarly, post-translational modification proteins such as chaperonins had lower expression (6/8) with the only exception of *groEL* and *groES*, which belong to close loci (B5T_03449 and B5T_03450, respectively, Table 2).

Upregulated COGs related with energy production and conversion were mainly associated with the F-type ATP synthase (Table 3). This enzymatic complex is divided into two major components, a central stalk and a stator, which are constituted by different subunits. All the gene loci related to this enzyme complex are closely located (B5T_04416 to B5T_04423, Table 3) but only 50% of them were upregulated (4/8): in particular genes related with the expression of the stator subunits (3/5) were upregulated or remained unaffected (2/5), indicating that functionality of this component is of primary importance under mild pressure. Despite respiration rates were not affected by increased pressure (Fig. 3b), cytochrome c (16/18) and b (2/2) related genes were downregulated at 10 MPa (Table 3), with the exception of ubiquinol-cytochrome c reductases genes the expression of which was upregulated (2/2, Table 3). Furthermore, we found that a pool of genes encoding for Na^+^ transporting NADH reductases (from B5T_01840 to B5T_01844) was slightly affected by pressure as transcription of 1/5 was enhanced (0.98, Table 3) and 2/5 remained unaffected, indicating a potential role of this enzyme complex under mild pressure.

Transcriptional functionality did not appear to be affected by mild pressure as all related genes were either unaffected or downregulated with few exceptions (Table 4). Genes encoding DNA repair proteins such as *ruvC*, a histone-like bacterial DNA repair and recombination protein and *recF* were among those with enhanced expression at 10 MPa, although the majority of these genes were generally downregulated (7/11, Table 4). Further, all transcriptional regulators were downregulated (7/9) with the exception of two *MarR* and *LysR* family genes (Table 4). Finally, we found that all genes related to the pathway involved in protein export and cell damage were unaffected (2/9) or downregulated (6/9), with the exception of the gene encoding for the YajC subunit (Table 4). Similarly, upregulation of genes related with the osmolite ectoine was not observed (Table 4). This was in agreement with the lack of ectoine detection in cells incubated at both 0.1 and 10 MPa (data not shown), thus confirming our previous observations on the structural resistance by the cells (Fig. 4). Finally, genes related to alkane activation, such as alkane hydroxylase (B5T_00103) or cytochrome P450 (B5T_02075), and fatty acids degradation were all strongly downregulated under mild pressure (Table 5).

## Discussion

Gene expression in high-pressure-adapted microbes from deep-sea has been elucidated over the last decades (reviewed in^20^). Description of pressure-sensitive pools of genes (the “so-called” *stimulons*^21^) and/or individual genes and gene traits revealed that they were regulated in several different microbes. The most notable ones include genes which code for heat or cold shock proteins (Hsp or Csp, respectively), outer membrane proteins (Omp), cell division proteins, ribosomes, elongation factors, cytochromes and DNA repair proteins (sigma factor family)^22^. Provided that the average depth of the Earth’s oceans is about 3800 m (corresponding to about 38 MPa) a widely shared hypothesis considers that pressure-loving bacteria (piezophiles and hyperpiezophiles) would possibly inhabit niches at this depth and further below^23^. Hence, the mechanisms involved in high-pressure-resistance such as *in vivo* protein synthesis have been reviewed for pressure ranges between 55 and 400 MPa^24^,^25^,^26^,^27^,^28^,^29^. On the contrary, little information on the effect of mild hydrostatic pressure on microbial metabolism is available^30^,^31^. While the study of piezophiles may reveal novel cellular mechanisms supporting life in the deep biosphere, the study of piezotolerant and piezosensitive bacteria may highlight the most sensitive pathways affected by hydrostatic pressure. Albright and Morita^32^ found that in the psychrophilic marine bacterium *Vibrio marinus* the rate of protein and RNA synthesis was affected after being exposed to 20 MPa for 60 min, although 1-atm rates were resumed shortly afterwards. Turley^33^ observed reduced uptake of ^3^H-thymidine and ^3^H-leucine for microbial protein synthesis between 0.1 and 43 MPa, but no effect on cell abundance.

In the present study, the hydrocarbon degraders *Alcanivorax dieselolei* KS_293 and *Alcanivorax jadensis* KS_339 showed a piezosensitive profile, with significant growth reduction already at 5 MPa (Fig. 1b), suggesting that their biodegradation potential may decrease with depth. However, under 10 MPa *A. dieselolei* KS_293 showed unaffected carbon degradation capacity (Fig. 3a), higher PO_4_^3−^ uptake (Fig. 3c) and lower number of damaged cells (Fig. 4) than *A. jadensis* KS_339, indicating a certain level of resistance to the stressing effects derived from hydrostatic pressure. Analysis of the transcriptome of *A. dieselolei* KS_293 revealed that expression of the large majority of its genome was downregulated at mild pressure (Fig. 5), while expression of only few pathways preserving critical functions, such as protein synthesis and energy production, was enhanced. Remarkably, most of the known pressure-responsive genes were not upregulated (Table S3) together with those involved in alkanes activation and fatty acids degradation (Table 5). These results suggest that bioremediation capacity of piezosensitive hydrocarbonoclastic bacteria may be already highly impaired at mild pressure, and that the interplay of the upregulated pathways has a role in counteracting mild pressure effects (Fig. 6).

One of the most affected functions in cells exposed to pressure is translation. *In vitro*, ribosomal proteins associated with mRNA and tRNA show improved stability and can resist up to 100 MPa, while uncharged ribosomes dissociated at 60 MPa^34^. Correlation between loss of cell viability and ribosome integrity at high pressure has been postulated^23^. In the present study, enhanced transcription of 30S and 50S ribosomal proteins was observed in *A. dieselolei* KS_293 when pressure was increased from 0.1 to 10 MPa (Table 1). Despite pressure reduced growth yields (Fig. 1b) cell viability was not affected (Fig. 4), indicating that cells division occurred at a slower pace. Limited growth has been proposed to be linked with impaired protein synthesis in microbes adapted to atmospheric pressure^35^,^36^.

One of the most pressure-sensitive steps affecting translation capacity is binding of aminoacyl-tRNA to ribosomes^26^ as it determines a conformational change leading to an increase in volume, the latter being an unfavored process under pressure^37^. In *A. dieselolei* KS_293, the majority of the translational elongation factors were either unaffected or upregulated (Table 2), in particular Tu and Ts, which have the role of binding the aminoacyl-tRNA to the A-site of the ribosome (Table 2). This further supports the hypothesis that translation capacity was affected, as also observed at much higher pressure (45 MPa) in short-term (30 min) experiments with *Lactobacillus sanfranciscensis*^38^.

However, expression of genes related to tRNA modifying proteins was among the most enhanced ones in the whole transcriptome (Table 2), as for *truB*. The latter expresses a pseudouridine synthase known for producing a pseudouridine in tRNAs, carrying RNA chaperone activity and assisting in the correct folding of tRNA, a process giving selective advantage (though not essential) for cell growth^39^. Translation accuracy was also potentially affected by pressure in *A. dieselolei* KS_293, as suggested by the upregulation of chaperonins such as GroEL and GroES (Table 2)^40^. The reason why only this type of chaperonins/small Hsp (Table 2 and S3) were upregulated under pressure remains unclear.

Adenosine triphosphate (ATP) is synthesized by the F-type ATP synthase (or F_1_F_0_ ATP synthase). This transmembrane, proton-force driven complex is known to be the smallest motor enzyme in nature, and uses a transmembrane electrochemical gradient of protons (or Na^+^ ions) to manufacture ATP from adenosine diphosphate (ADP) and inorganic PO_4_^3− ^41. It is constituted by a central stalk (made of gamma and epsilon subunits in the F_1_ and c subunit ring in the F_0_ part), which rotates relative to a stator (composed of alpha, beta, delta, a and b subunits^42^) during ATP hydrolysis and synthesis. Genes related with the stator were either unaffected or upregulated in *A. dieselolei* KS_293 (Table 3) suggesting that functionality of this transmembrane unit was affected at mild pressure. Upregulation of these genes may have been linked to enhanced ATP generation necessary for coping with sustained cell maintenance when pressure increased. Previous reports revealed that unbalanced ATP generation and demand in *Streptococcus faecalis* cells exposed to 408 atm (equivalent to 40.8 MPa) actually reduced growth rates, but that pressure-volume work, turnover of proteins, peptidoglycan, or stable RNA were not related with the use of ATP^43^. However, we did not observe higher PO_4_^3−^ consumption per cell (Fig. 3c), cell integrity was negligibly affected (Fig. 4), production of osmolites was not activated (Table 4) and genes related with protein export and cell maintenance were only marginally affected by pressure variation (Table 4). While ATP generation was potentially impacted, upregulation of genes related only to the stator could be attributed to structural problems that this transmembrane component faced under 10 MPa.

Microbes dealing with temperature variations optimize ion permeability, such as proton translocation and ATP synthesis, for bio-energetic purposes^44^. We found that the pool of genes expressing the Na^+^ -translocating NADH reductase complex (RNF-NQR) was slightly affected under mild pressure (Table 3). NADH can be oxidized by the respiratory chain of bacteria *via* NADH:quinone oxidoreductases that belong to 3 distinct enzyme families: NDH-1, NDH-2, and NQR^45^. The NQR-type enzymes are Na^+^ -motive NADH-quinone oxidoreductases consisting of 6 subunits and several cofactors. The NADH-quinone oxidoreductase activity of these enzymes is stimulated by Na^+^ ions and is coupled with pumping of Na^+^ rather than H^+^. As explained by van de Vossenberg *et al*.^44^, the proton motive force in bacteria dealing with temperature changes is sustained by increasing the rate of proton pumping, by altering the membrane composition to reduce permeability to ions, or by coupling proton-driven processes to less permeable ions such as Na^+^. A similar process has been proposed as alternative respiration pathway in *Shewanella benthica* grown under 60 MPa^46^,^47^. In particular, a NADH-oxidoreductase would reduce a quinone and this will reduce a quinone oxidase, which in turn would use O_2_ as the final electron acceptor. This pathway would not need cytochromes c and b as observed in *A. dieselolei* KS_293, where these genes were downregulated, and would rather use quinone-oxidoreductases, which are mostly upregulated under mild pressure (Table 3). By preferring the quinol-oxidoreductase pathway bacteria are known to maintain efficient respiration, which indeed in the present study was not affected at higher pressure than atmospheric (Fig. 3b).

Surface waters and deep sea are two distinguished niches divided by a large area, the mesopelagic zone, where hydrostatic pressure critically increases within short depths. The metabolic functions of piezosensitive bacteria may result influenced as an attempt to survive in this zone. Response to mild pressure increase differentially impacts hydrocarbonoclastic isolates belonging to the same genus. In the structurally resistant *A. dieselolei* KS_293 growth rates appear to be lowered by the interplay between impaired protein synthesis and energy production. Furthermore, normal respiration pathways at atmospheric pressure may be affected already under 10 MPa. The response to these critical steps potentially shapes the structure of microbial communities developed after oil spills at sea, therefore playing a role in successful bioremediation strategies.

## Materials and Methods

### Strains, culture media and growth conditions

*Alcanivorax jadensis* KS_339 and *Alcanivorax dieselolei* KS_293 were isolated from surface waters collected in the Gibraltar Strait and the Levantine basin (35° 54′, −7° 00′ and 34° 04′, 34° 00′, respectively) performing enrichment cultures on ONR7a medium^48^ supplemented with 1% (v:v) diesel oil as sole carbon source.

After isolation, strains were axenically cultivated in static glass bottles of 250 mL (operating volume 100 mL), using ONR7a medium, at initial pH 7.6, for 4 to 7 days at room temperature, providing n-dodecane (Sigma Aldrich, Belgium) 1% (v:v) as sole carbon source.

### Mild hydrostatic pressure experiments

Growing cultures were collected, centrifuged at 4000 rpm for 10 min at 4 °C (Sorval RC5c PLUS, Beckman, Suarlée, Belgium) and resuspended in fresh ONR7a medium at initial optical density (OD_610_) of 0.100. Then, 3.5 mL of liquid culture suspension was transferred into sterile 10 mL syringes. Gas phase (equal to 6.5 mL) was constituted of air, which provided O_2_ to the cells during the subsequent incubation. n-dodecane (C12) 1% (v:v) was supplied as the sole carbon source. Syringes were closed using a sterile three-way valve, and placed in a 1 L T316 stainless steel high-pressure reactor (HPR) (Parr, USA). Reactor was filled with deionized water and hydrostatic pressure was increased up to 5 or 10 MPa by pumping water with a high-pressure pump (HPLC pump series III, SSI, USA). Pressure was transmitted to the cultures through the piston of the syringe. Experiments at atmospheric pressure (0.1 MPa) were run adjacent to the HPR. Control experiments were constituted by sterile syringes supplied only with sterile medium. Reactors were incubated in a temperature-controlled room at 20 °C for 4 days. At the end of the experiments, pressure was gently released up to 0.1 MPa and syringes were set aside for 30–60 min before running analyses.

Control experiments were conducted under ambient pressure using the same set up described above, with the only exception that air was substituted with pure, sterile O_2_ (Linde, Schiedam, The Netherlands). Both strains *A. jadensis* KS_339 and *A. dieselolei* KS_293 were tested in 3 independent replicates).

### Microbial analyses

Optical density was measured at 610 nm with a spectrophotometer (Isis 9000, Dr Lange, Germany). Cell count and intact/damaged cell count was performed by flow cytometry. SYBR® Green I and Propidium Iodide (PI) were used in combination to discriminate cells with intact and damaged cytoplasmic membranes^49^,^50^. Staining solution was prepared as follows: PI (20 mM in dimethyl sulfoxide [DMSO], LIVE/DEAD BacLight Kit, Invitrogen, Belgium) was diluted 50 times and SYBR® Green I (10,000 times concentrate in DMSO, Invitrogen) was diluted 100 times in 0.22 μm-filtered-DMSO. Water samples were stained with 10 μL/mL staining solution and 10 μL/mL EDTA (pH 8, 500 mM) for outer membrane permeabilization. Before staining, samples of 1 mL were maintained at room temperature for 30 min to minimize staining temperature effect. Prior to flow cytometric analysis, stained samples were incubated for 13 min in the dark at 37 °C.

Flow cytometry was performed using a CyAn™ ADP LX flow cytometer (Dakocytomation, Heverlee, Belgium) equipped with a 50-mW Sapphire solid-state diode laser (488 nm). Stability and performance was performed using the Cyto-Cal Alignment Beads and Cyto-Cal multifluor Fluorescent Intensity Calibrator (Distrilab, Leusden, The Netherlands). Green and red fluorescence were collected with photomultiplier tubes using 530/40 and 613/20 bandpass filters respectively. Forward (FS) and side light scatter (SS) were collected with a 488/10 bandpass filter. Milli-Q water was used as the sheath fluid. All samples were collected as logarithmic signals triggered on the green fluorescence channel. Data for 20,000 events for each sample run was collected.

### Chemical analyses

O_2_ respiration and CO_2_ production were assessed by comparing the head space biogas composition of syringes inoculated with *Alcanivorax* species and sterile controls at the end of the incubation. Gas phase was analyzed with a Compact GC (Global Analyser Solutions, Breda, The Netherlands), equipped with a Molsieve 5 A pre-column and two channels. In channel 1, a Porabond column detected CH_4_, O_2_, H_2_ and N_2_. In channel 2, a Rt-Q-bond pre-column and column detected CO_2_, N_2_O and H_2_S. Concentrations of gases were determined with a thermal conductivity detector. pH was measured using a pH meter (Herisau, Metrohm, Switzerland). Sulphate and phosphate were quantified with a Compact Ion Chromatograph (Herisau, Metrohm, Switzerland) equipped with a conductivity detector. Dodecane concentration was evaluated using a GC equipped with a flame ionized detector (FID) (Agilent Technologies, Santa Clara, USA), equipped with a HP-5 capillary column (30 m; 0.25 mm), following these conditions: initial temperature, 60 °C; isothermal for 1 min; temperature rate, 10 °C min^−1^; final temperature, 320 °C; isothermal for 5 min. The injector (splitless mode) was at 270 °C, FID at 320 °C; carrier gas (N_2_) flow rate was 60 mL min^−1^ and injected sample volume was 1 μL. First, 0.7 mL of culture were removed from syringes and extracted from the water-phase using 1:1 n-hexane. Samples were vigorously shaken for 1 min and set aside for 15 min. The upper layer of hexane and extracted dodecane was collected and injected into the GC-FID.

### Transcriptomic analysis in A. dieselolei KS_293

Ten independent cultures of *A. dieselolei* KS_293 were grown at both 0.1 and 10 MPa as described above. At the end of the experiments, pressure was gently released and cultures pooled together in order to have enough cell material and to average the response to pressure in independently grown cultures. Cells were recovered by centrifugation at 4 °C for 5 min at 13000 rpm (Sorval RC5c PLUS, Beckman, Suarlée, Belgium) and pellets stored at −80 °C for further RNA extraction.

#### RNA extraction and QC

RNA was isolated from pelleted cells using the Rneasy Mini Kit (Qiagen, Antwerp, Belgium) following manufacturer’s instructions. On-column DNase digestion was performed during RNA extraction. RNA concentration was determined using the NanoDrop 2000 UV-Vis spectrophotometer (Thermo Scientific, Waltham, MA, USA). Pellets originated from at 0.1 and 10 MPa had 615.2 and 311.5 ng/μL, respectively. RNA quality control was performed using the 2100 Bioanalyzer microfluidic gel electrophoresis system (Agilent Technologies, Santa Clara, USA).

#### RNA library prep and sequencing

Libraries for RNA-sequencing were prepared using the ScriptSeq Complete (Bacteria) sample prep kit (Epicentre – Illumina, San Diego, CA, USA). Starting material (1000 ng) of total RNA was depleted of rRNAs using Ribo-Zero magnetic bead based capture-probe system (Illumina, Hayward, USA). Remaining RNA (including mRNAs, lin-cRNAs and other RNA species) was subsequently purified (Agencourt RNA- Clean XP, Beckman Coulter, Brea, CA, USA) and fragmented using enzymatic fragmentation. First strand synthesis and second strand synthesis were performed and double stranded cDNA was purified (AgencourtAMPure XP, Beckman Coulter). RNA stranded libraries were pre-amplified and purified (AgencourtAMPure XP, Beckman Coulter). Library size distribution was validated and quality inspected using the 2100 Bioanalyzer (high sensitivity DNA chip, Agilent Technologies). High quality libraries were quantified using the Qubit Fluorometer (Life Technologies, Carlsbad, CA, USA), concentration normalized and samples pooled according to number of reads. Sequencing was performed on a NextSeq500 instrument using Mid Output sequencing kit (150 cycles) according to manufacturer’s instructions (Illumina, Hayward, USA).

#### Data processing workflow

Data analysis pipeline was based on the Tuxedo software package (Oracle, Redwood Shores, CA, USA). Components of the RNA-seq analysis pipeline included Bowtie2 (v. 2.2.2), TopHat (v2.0.11) and Cufflinks (v2.2.1) and are described in detail below. TopHat is a fast splice junction mapper for RNA-Seq reads, which aligns sequencing reads to the reference genome using the sequence aligner Bowtie2. TopHat uses sequence alignments to identify splice junctions for both known and novel transcripts. Cufflinks takes the alignment results from TopHat to assemble the aligned sequences into transcripts, constructing a map of the transcriptome. A previously reported transcript annotation was used to guide the assembly process^51^.

#### Data analysis

Genes were grouped according to orthologous clusters using the database provided by Ortholuge DB^52^. Only clusters classified as supporting-species-divergence (SSD) were considered and the rest were discarded (Borderline-SSD, Divergent-SSD, Similar Non-SSD and unevaluated orthologs [RBB]). Up and downregulation analysis was expressed on a log2 basis, indicating fold changes in fragments per kilobase of transcript per million mapped reads (FPKM) between samples at 0.1 and 10 MPa. Gene clusters were arbitrarily considered up-regulated at mild pressure when their log2 fold change was higher than 0.5 between 0.1 and 10 MPa. On the contrary, it was considered that downregulated genes had a −0.50 fold change between the same range of pressures. All the gene clusters that were expressed between −0.5 and 0.5 were considered to be unaffected by the increase in pressure. Hence, the ±0.5 log2 fold change was established in order to have a reasonable compromise in the definition of both upregulated and unaffected genes, provided that a higher threshold may be more appropriate to assess upregulation but would result into an over estimation of unaffected genes. Final analysis of up and down-regulated genes, and clusters of orthologous gene (COG) category was done using the database provided by KEGG (www.genome.jp/kegg).

### Statistical analysis

Results were expressed as mean values of experiments made in 4 to 20 independent replicates. Bars in the graphs indicate a 95% confidence interval (95% CI) calculated using a Student *t*-test with a two-sided distribution. Statistical significance was assessed using a nonparametric test (Mann–Whitney test) which considered a two-sided distribution with 95% CI.

## Additional Information

**How to cite this article**: Scoma, A. *et al*. Microbial oil-degradation under mild hydrostatic pressure (10MPa): which pathways are impacted in piezosensitive hydrocarbonoclastic bacteria? *Sci. Rep*. **6**, 23526; doi: 10.1038/srep23526 (2016).

## Supplementary Material

Supplementary Information

## Figures and Tables

**Table 1:** Ribosomal protein gene expression in *A. dieselolei* KS_293 cells under 10 and 0.1 MPa.

Regulation	log2 FC	10 MPa	0.1 MPa	Cluster ID	Locus Tag	Description
+	4.47	2536.9	114.3	1929	B5T_03774	50S ribosomal protein L36
+	4.12	120.0	6.9	913	B5T_03782	30S ribosomal protein S14
+	2.52	8358.9	1461.7	334	B5T_03777	50S ribosomal protein L30
+	2.46	2871.8	520.6	1912	B5T_03790	50S ribosomal protein L22
+	2.35	27.9	5.5	1800	B5T_03806	50S ribosomal protein L11
+	2.19	809.0	177.3	1335	B5T_03781	30S ribosomal protein S8
+	2.17	1256.4	278.6	66	B5T_03786	30S ribosomal protein S17
+	2.11	199.9	46.4	2176	B5T_03787	50S ribosomal protein L29
+	1.94	651.8	169.3	808	B5T_01420	50S ribosomal protein L19
+	1.81	117.7	33.5	2150	B5T_03778	30S ribosomal protein S5
+	1.58	287.3	96.2	1378	B5T_03804	50S ribosomal protein L10
+	1.55	207.0	70.6	1281	B5T_03780	50S ribosomal protein L6
+	1.53	201.4	69.7	180	B5T_03783	50S ribosomal protein L5
+	1.49	42.2	15.1	2106	B5T_03779	50S ribosomal protein L18
+	1.42	3469.9	1300.6	1903	B5T_03803	50S ribosomal protein L7/L12
+	1.19	49.2	21.6	1232	B5T_02734	50S ribosomal protein L32
+	1.19	293.9	128.8	906	B5T_03784	50S ribosomal protein L24
+	1.16	113.4	50.9	662	B5T_03788	50S ribosomal protein L16
+	1.12	42.4	19.6	1059	B5T_03776	50S ribosomal protein L15
+	1.1	302.2	140.7	1541	B5T_03789	30S ribosomal protein S3
+	1.03	380.9	186.7	594	B5T_03987	50S ribosomal protein L31
+	0.96	47.0	24.2	1225	B5T_03769	50S ribosomal protein L17
+	0.92	1029.5	543.5	2053	B5T_03202	50S ribosomal protein L35
+	0.88	193.8	105.1	327	B5T_03728	50S ribosomal protein L27
+	0.75	566.0	335.5	1101	B5T_00191	50S ribosomal protein L28
+	0.73	228.6	137.9	1759	B5T_03771	30S ribosomal protein S4
+	0.72	734.2	446.1	848	B5T_03521	50S ribosomal protein L13
+	0.69	190.8	118.4	1786	B5T_03855	30S ribosomal protein S15
						
=	0.42	134.5	100.4	251	B5T_03794	50S ribosomal protein L4
=	0.35	279.4	218.7	1161	B5T_03792	50S ribosomal protein L2
=	0.35	209.7	164.1	94	B5T_03799	30S ribosomal protein S7
=	0.23	16.1	13.7	1356	B5T_03520	30S ribosomal protein S9
=	0.18	764.7	672.8	258	B5T_01417	30S ribosomal protein S16
=	0.17	107.1	94.9	1132	B5T_03725	30S ribosomal protein S20
=	0.1	455.0	425.9	575	B5T_02974	30S ribosomal protein S1
=	0.1	176.3	165.0	1111	B5T_03791	30S ribosomal protein S19
=	0.08	91.1	86.4	151	B5T_03793	50S ribosomal protein L23
=	0.08	103.4	97.9	263	B5T_01921	30S ribosomal protein S2
=	0.05	240.4	231.7	917	B5T_00790	30S ribosomal protein S6
=	0.02	443.1	436.6	665	B5T_00192	50S ribosomal protein L33
=	−0.17	36.4	40.8	1365	B5T_00964	30S ribosomal protein S21
=	−0.2	255.5	293.0	1254	B5T_03772	30S ribosomal protein S11
=	−0.22	772.1	899.1	466	B5T_03796	30S ribosomal protein S10
=	−0.31	962.3	1193.7	1397	B5T_03795	50S ribosomal protein L3
=	−0.44	11.7	15.9	610	B5T_03805	50S ribosomal protein L1
=	−0.46	4.2	5.8	1385	B5T_00793	50S ribosomal protein L9
						
−	−0.53	45.1	65.1	2091	B5T_03201	50S ribosomal protein L20
−	−0.73	595.5	985.4	1647	B5T_00792	30S ribosomal protein S18
−	−0.79	82.7	143.3	1895	B5T_03773	30S ribosomal protein S13
−	−0.8	81.8	142.0	113	B5T_03866	Ribosomal RNA large subunit methyltransferase E
−	−0.9	41.0	76.6	1244	B5T_03634	50S ribosomal protein L25
−	−0.92	7.4	14.1	489	B5T_01980	Ribosomal large subunit pseudouridine synthase E
−	−1.12	19.0	41.3	2097	B5T_03496	Ribosomal RNA small subunit methyltransferase H
−	−1.25	9.0	21.6	1977	B5T_04427	Ribosomal RNA small subunit methyltransferase G
−	−1.27	9.5	22.9	2158	B5T_03261	Ribosomal protein S6 modification protein
−	−1.44	4.8	13.0	645	B5T_00115	Ribosomal RNA small subunit methyltransferase B
−	−1.92	20.9	79.0	518	B5T_01191	Ribosomal RNA large subunit methyltransferase N
−	−1.94	3.1	11.8	915	B5T_03262	Ribosomal protein S6 modification protein
−	−2.07	39.0	163.4	2029	B5T_03800	30S ribosomal protein S12
−	−2.12	549.9	2393.9	474	B5T_03578	Ribosomal subunit interface protein
−	−2.12	28.3	122.8	1883	B5T_01013	Ribosomal protein L11 methyltransferase
−	−2.6	1.4	8.5	233	B5T_02829	Ribosomal RNA large subunit methyltransferase M
−	−3.04	6.5	53.9	889	B5T_00978	Ribosomal RNA small subunit methyltransferase A
−	−3.14	1.2	10.3	895	B5T_01813	Ribosomal RNA large subunit methyltransferase L
−	−3.31	0.3	3.2	1702	B5T_03442	Ribosomal-protein-alanine acetyltransferase
−	−4.28	2.2	41.6	2014	B5T_01081	Ribosomal RNA large subunit methyltransferase H

**Table 2:** Expression of tRNA modification and translation elongation factors, and chaperonin proteins genes in *A. dieselolei* KS_293 cells under 10 and 0.1 MPa.

Function	Regulation	log2 FC	10 MPa	0.1 MPa	Cluster ID	Locus Tag	Description
tRNA modification							
	+	3.09	231.6	27.2	789	B5T_03856	tRNA pseudouridine synthase B; truB
	+	1.45	349.7	127.6	718	B5T_01185	Iron-binding protein IscA; thiolation factor
Translation elongation factors							
	+	0.54	298.1	205.6	155	B5T_03797	Elongation factor Tu; tuf
	+	0.54	74.4	51.2	930	B5T_01922	Elongation factor Ts, tsf
	=	0.43	302.0	224.3	155	B5T_03809	Elongation factor Tu; tuf
	=	0.17	89.0	79.3	1100	B5T_03798	Elongation factor G, fusA
	=	−0.13	100.3	109.6	573	B5T_00690	Elongation factor P; efp
	−	−2.3	15.5	76.0	453	B5T_01535	Elongation factor 4; lepA
Chaperonin proteins							
	+	0.62	731.7	477.7	920	B5T_03449	60 kDa chaperonin, groEL
	+	0.59	1091.2	725.0	1897	B5T_03450	10 kDa chaperonin, groES
	−	−0.65	38.5	60.2	572	B5T_02668	Chaperone protein htpG
	−	−1.04	39.6	81.4	342	B5T_03872	Chaperone protein dnaJ
	−	−1.22	506.6	1180.5	2001	B5T_03873	Chaperone protein dnaK
	−	−1.59	11.1	33.3	1599	B5T_03874	Protein grpE
	−	−2.05	150.9	623.4	104	B5T_03703	ATP-dependent Clp protease, ATP-binding subunit; ClpB
	−	−3.25	0.3	2.8	315	B5T_00544	33 kDa chaperonin, hslO

**Table 3:** Expression of some energy production and conversion COG in *A. dieselolei* KS_293 cells under 10 and 0.1 MPa.

Function	Regulation	log2 FC	10 MPa	0.1 MPa	Cluster ID	Locus Tag	Description
ATP synthase							
							Central stalk
	+	0.64	107.8	69.3	485	B5T_04418	ATP synthase gamma chain
	−	−1.22	31.4	73.3	1610	B5T_04422	ATP synthase subunit c 1
	−	−1.63	51.8	159.9	1581	B5T_04416	ATP synthase epsilon chain
							Stator
	+	1.05	153.4	74.3	942	B5T_04420	ATP synthase subunit delta
	+	1	81.6	40.7	506	B5T_04421	ATP synthase subunit b
	+	0.7	755.7	465.8	323	B5T_04423	ATP synthase subunit a
	=	0.37	41.2	31.8	163	B5T_04419	ATP synthase subunit alpha
	=	−0.34	101.4	128.3	1566	B5T_04417	ATP synthase F1, beta subunit
Cytochrome c							
	=	0.27	29.4	24.4	288	B5T_04195	Cytochrome c subfamily, putative
	=	−0.21	120.1	138.7	1326	B5T_04288	Cytochrome c-type protein
	−	−0.91	7.8	14.7	1440	B5T_04287	Cytochrome c subfamily, putative
	−	−1.02	63.0	127.8	1533	B5T_01136	Cytochrome c oxidase assembly protein CtaG/Cox11
	−	−1.24	21.7	51.2	1852	B5T_01137	Cytochrome c oxidase, subunit I
	−	−2.53	13.0	75.2	386	B5T_01576	Cytochrome c-type biogenesis protein CcmE
	−	−2.6	8.1	49.1	947	B5T_01135	Cytochrome c oxidase, subunit III
	−	−2.61	15.8	96.3	1875	B5T_02284	Cytochrome c oxidase, cbb3-type, subunit II
	−	−2.62	8.8	54.0	110	B5T_02282	Cytochrome c oxidase, cbb3-type, subunit III, putative
	−	−2.69	11.1	72.0	2031	B5T_02615	Cytochrome c subfamily, putative
	−	−2.89	1.9	13.9	1575	B5T_01415	Cytochrome c assembly protein
	−	−3.03	2.2	18.0	1166	B5T_01572	Cytochrome c biogenesis ATP-binding export protein CcmA
	−	−3.07	2.4	19.8	1142	B5T_01138	Cytochrome c oxidase subunit 2
	−	−3.12	9.5	82.7	1284	B5T_02285	Cytochrome c oxidase, cbb3-type, subunit I
	−	−3.45	3.1	34.3	862	B5T_02281	Cytochrome c oxidase accessory protein CcoG
	−	−3.87	0.4	5.5	611	B5T_01554	Cytochrome c heme lyase subunit CcmL
	−	−4.14	0.8	13.3	421	B5T_01552	Cytochrome c-type biogenesis protein CcmF
	−	−5.07	0.4	11.7	1861	B5T_01019	Cytochrome c biogenesis protein transmembrane region
Ubiquinol-Cytochrome c							
	+	0.79	215.1	124.4	1648	B5T_03519	ubiquinol-cytochrome c reductase iron-sulfur subunit; petA
	+	0.58	164.9	110.0	1172	B5T_03517	ubiquinol-cytochrome c reductase cytochrome c1 subunit; petC
	−	−2.48	18.2	101.9	1213	B5T_00990	ubiquinol oxidase, subunit II, putative
Cytochrome b							
	−	−0.97	25.4	49.7	285	B5T_03518	cytochrome b; petB
	−	−0.58	102.7	153.2	1237	B5T_02687	succinate dehydrogenase, cytochrome b556 subunit
Na+ translocating NADH reductase							
	+	0.98	51.8	26.3	781	B5T_01844	Na+-translocating NADH-quinone reductase subunit E
	=	−0.32	52.3	65.5	615	B5T_01843	Na+-translocating NADH-quinone reductase subunit D
	=	−0.33	14.8	18.6	359	B5T_01842	Na+-transporting NADH-ubiquinone oxidoreductase subunit C
	−	−0.52	31.2	44.6	2010	B5T_01840	Na+-translocating NADH-quinone reductase subunit A
	−	−0.78	24.4	42.0	1158	B5T_01841	Na+-translocating NADH-ubiquinone oxidoreductase subunit B

**Table 4:** Expression of genes related with DNA processing, protein export and osmolites production in *A. dieselolei* KS_293 cells under 10 and 0.1 MPa.

Function	Regulation	log2 FC	10 MPa	0.1 MPa	Cluster ID	Locus Tag	Description
RNA polymerases							
	+	0.54	22.8	15.7	497	B5T_00166	DNA-directed RNA polymerase subunit omega
	=	0.35	414.0	325.0	739	B5T_03770	DNA-directed RNA polymerase, alpha subunit
	=	−0.02	80.8	81.9	1558	B5T_03801	DNA-directed RNA polymerase subunit beta'
	=	−0.3	54.8	67.5	1782	B5T_03802	DNA-directed RNA polymerase subunit beta
DNA repairing system							
	+	1.44	3.0	1.1	1577	B5T_03233	Crossover junction endodeoxyribonuclease; ruvC
	+	0.78	495.3	287.9	1089	B5T_02378	Histone-like bacterial DNA-binding protein
	+	0.76	34.2	20.3	366	B5T_00003	DNA replication and repair protein; recF
	=	−0.49	12.3	17.3	1956	B5T_03101	Recombinase A; recA
	−	−2.78	3.6	24.4	324	B5T_03876	DNA repair protein; recN
	−	−2.82	0.6	4.0	1043	B5T_03100	Regulatory protein; recX
	−	−2.96	2.4	18.4	1708	B5T_03007	Recombination protein; recR
	−	−3.36	2.2	22.1	393	B5T_03250	ATP-dependent DNA helicase; recQ
	−	−3.44	0.9	10.1	478	B5T_00150	ATP-dependent DNA helicase; recG
	−	−4.34	0.9	17.7	48	B5T_01518	Single-stranded-DNA-specific exonuclease; recJ
	−	−4.38	0.4	7.8	1179	B5T_01540	DNA repair protein; recO
Transcriptional regulators							
	+	0.71	44.1	26.9	1768	B5T_00887	Transcriptional regulator, MarR family
	+	0.65	2.8	1.8	275	B5T_02642	*LysR family transcriptional regulator*
	−	−3.87	7.8	114.2	891	B5T_02527	*TraR/DksA family transcriptional regulator*
	−	−4.25	1.8	33.7	200	B5T_04299	Transcriptional regulator, AraC family protein
	−	−4.34	1.5	30.8	1292	B5T_01803	Transcriptional regulator, TetR family
	−	−4.45	0.2	4.2	736	B5T_01483	Two component transcriptional regulator, winged helix family
	−	−4.48	0.1	3.2	1969	B5T_02708	putative transcriptional regulator
	−	−5.41	0.3	11.6	1735	B5T_01045	Two-component transcriptional regulator, LuxR family protein
	−	−5.44	0.3	12.3	1975	B5T_00817	transcriptional regulator; NrdR
Transcription elongation factors							
	=	0.1	41.0	38.3	680	B5T_03868	Transcription elongation factor greA 1; greA
	−	−0.52	114.6	164.3	2066	B5T_03859	Transcription elongation factor; nusA
	−	−2.2	14.5	66.3	585	B5T_04038	Transcription elongation factor greA 1; greB
Protein export							
	+	1.05	274.8	132.8	1399	B5T_03676	Preprotein translocase, YajC subunit
	=	0.25	296.8	250.3	869	B5T_03775	Preprotein translocase SecY subunit
	=	0.03	122.9	120.0	292	B5T_00612	Protein-export protein secB
	−	−0.54	33.6	48.8	1233	B5T_03808	Preprotein translocase subunit secE
	−	−0.65	28.5	44.6	2024	B5T_03861	Preprotein translocase SecG subunit, putative
	−	−1	55.9	111.9	1490	B5T_03674	Preprotein translocase SecF subunit
	−	−1.55	116.2	339.2	968	B5T_03479	Protein translocase subunit secA
	−	−1.97	12.1	47.5	821	B5T_04447	Membrane protein oxaA yidC
	−	−3.01	4.2	33.5	1027	B5T_03675	Preprotein translocase SecD subunit
Ectoine synthesis							
	=	−0.23	115.6	135.6	471	B5T_00884	Ectoine synthase
	−	−2.68	0.8	5.0	445	B5T_04304	Ectoine hydroxylase

**Table 5:** Expression of genes related with activation of alkanes and fatty acids degradation in *A. dieselolei* KS_293 cells under 10 and 0.1 MPa.

Function	Regulation	log2 FC	10 MPa	0.1 MPa	Cluster ID	Locus Tag	Description
Alkanes activation							
	−	−1.37	1066.6	2749.9	764	B5T_02075	Cytochrome P450 alkane hydroxylase
	−	−2.39	31.4	164.6	407	B5T_00103	Alkane hydroxylase
Fatty acids degradation							
	−	−0.62	77.7	119.0	1313	B5T_01517	3-hydroxyacyl-CoA dehydrogenase/short chain enoyl-CoA hydratase
	−	−0.88	40.8	75.1	898	B5T_00925	Acyl-CoA dehydrogenase
	−	−1.04	16.6	34.1	1411	B5T_00923	Acyl-CoA dehydrogenase
	−	−1.17	12.9	29.0	143	B5T_01516	3-ketoacyl-CoA thiolase
	−	−1.2	11.6	26.8	644	B5T_03556	Acyl-CoA dehydrogenase
	−	−1.34	12.6	32.0	660	B5T_02905	3-hydroxyacyl-CoA dehydrogenase
	−	−1.59	15.1	45.6	689	B5T_01467	Acyl-CoA dehydrogenase
	−	−1.98	1.4	5.5	1976	B5T_04210	Putative acetyl-CoA acetyltransferase with thiolase domain
	−	−2.09	3.3	14.2	1467	B5T_01622	Acyl-CoA dehydrogenase
	−	−2.39	5.0	26.5	858	B5T_01799	Acyl-CoA dehydrogenase
	−	−2.49	4.8	26.8	55	B5T_03005	Acyl-CoA dehydrogenase
	−	−2.5	423.6	2388.1	914	B5T_02077	Pyridine nucleotide-disulfide oxidoreductase domain protein
	−	−2.89	3.3	24.2	202	B5T_02439	Acyl-CoA dehydrogenase
	−	−2.95	1.3	9.7	654	B5T_01468	Acyl-CoA dehydrogenase
