# Supplementary material for: Microbial oil-degradation under mild hydrostatic pressure (10 MPa): which pathways are impacted in piezosensitive hydrocarbonoclastic bacteria?
Source: Sci Rep. 2016 Mar 29;6:23526. doi: 10.1038/srep23526 (PMC4810429; doi:10.1038/srep23526)
Supplement: Supplementary Information [file srep23526-s1.pdf]

Microbial oil-degradation under mild hydrostatic pressure (10MPa): which pathways are impacted in piezosensitive hydrocarbonoclastic bacteria?

Alberto Scoma[1+], Marta Barbato[2+], Emma Hernandez-Sanabria[1], Francesca Mapelli[2], Daniele Daffonchio[2,3], Sara Borin[2], and Nico Boon[1*]

[1] Laboratory of Microbial Ecology and Technology (LabMET), University of Gent, Coupure Links 653, B 9000 Gent, Belgium

[2] Department of Food, Environmental and Nutritional Sciences (DeFENS), University of Milano, Via Celoria 2, 20133, Milano, Italy

[3] Biological and Environmental Sciences and Engineering Division, King Abdullah University of Science and Technology (KAUST), 4700, Thuwal 23955-6900, Kingdom of Saudi Arabia

* corresponding author: Prof. Nico Boon, LabMET – Ugent, Coupure Links 653, 9000, Gent; Nico.Boon@UGent.be; Phone +32 (0)9 264 59 76; Fax +32 (0)9 264 62 48.

[+] these authors contributed equally to this work

Supplementary Information

Figure S1 C12 bioavailability in *A. jadensis* KS_339 (purple) and *A. dieselolei* KS_293 (green) under atmospheric (0.1 MPa) and mild pressure (5 and 10MPa). Initial C12 concentration provided was equal to 7.5 g L^{-1}. Bars indicate 95% confidence intervals.

Figure S2 Growth of *A. jadensis* KS_339 (purple) and *A. dieselolei* KS_293 (green) under atmospheric pressure (0.1 MPa) using air or pure O_2 as gas phase. Bars indicate 95% confidence intervals.

Figure S3 Total CO_2 production in *A. jadensis* KS_339 (purple) and *A. dieselolei* KS_293 (green) under atmospheric (0.1 MPa) and mild pressure (5 and 10MPa). Bars indicate 95% confidence intervals.

Table S1 Total amount of O_2 transferred to the liquid phase by increased hydrostatic pressure to 10MPa. Measurements were conducted with sterile controls at 10MPa. Provided that diffusion is linear[53], at 5MPa O_2 delivered to the culture was equal to 3.09mM.

Table S2 Respiration capacity of *Alcanivorax* cells under 5 and 10MPa. Cell number and respiration rate values are derived from Fig. 1B and 3B

Table S3 Expression of some typical pressure-resistance pool of genes in *A. dieselolei* KS_293 cells under 10 and 0.1MPa.

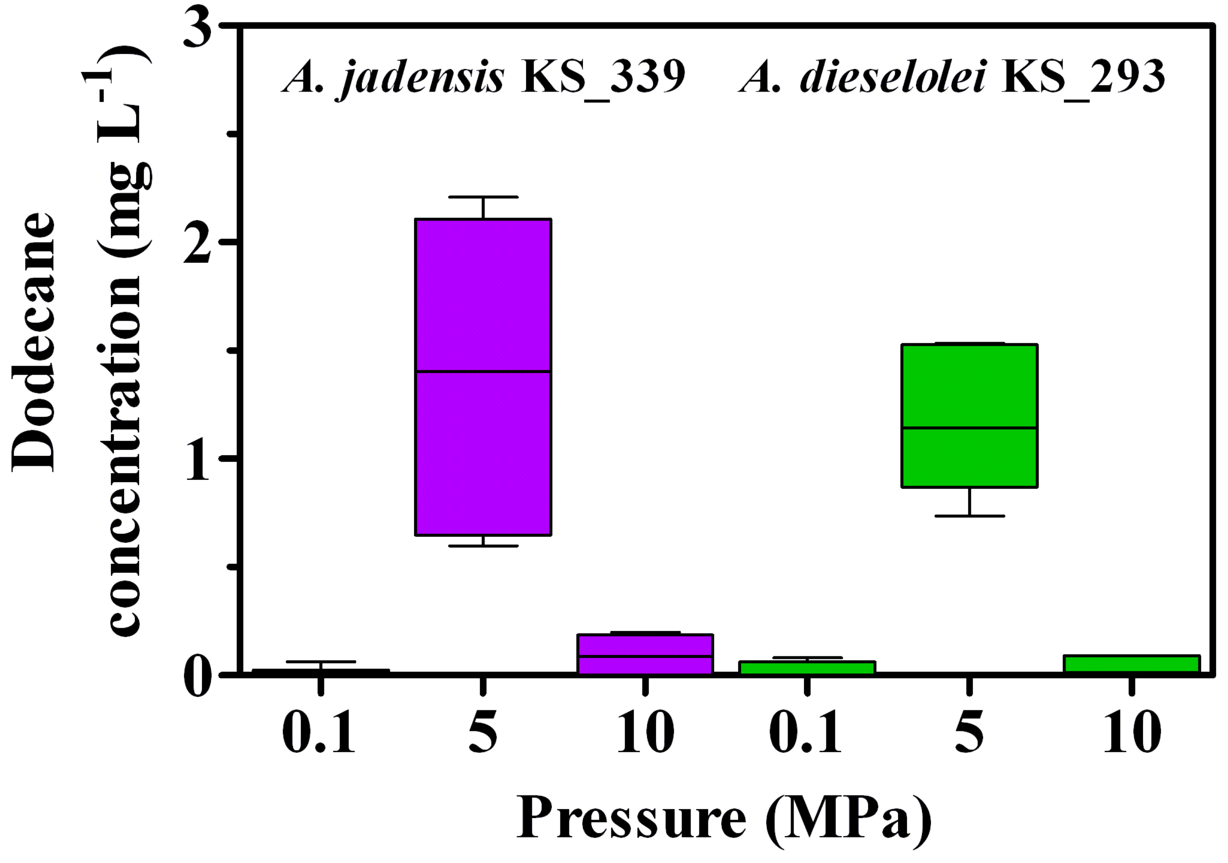

A. jadensis KS_339
A. dieselolei KS_293
Dodecane concentration (mg L-1)
0
1
2
3
0.1
5
10
0.1
5
10
Pressure (MPa)

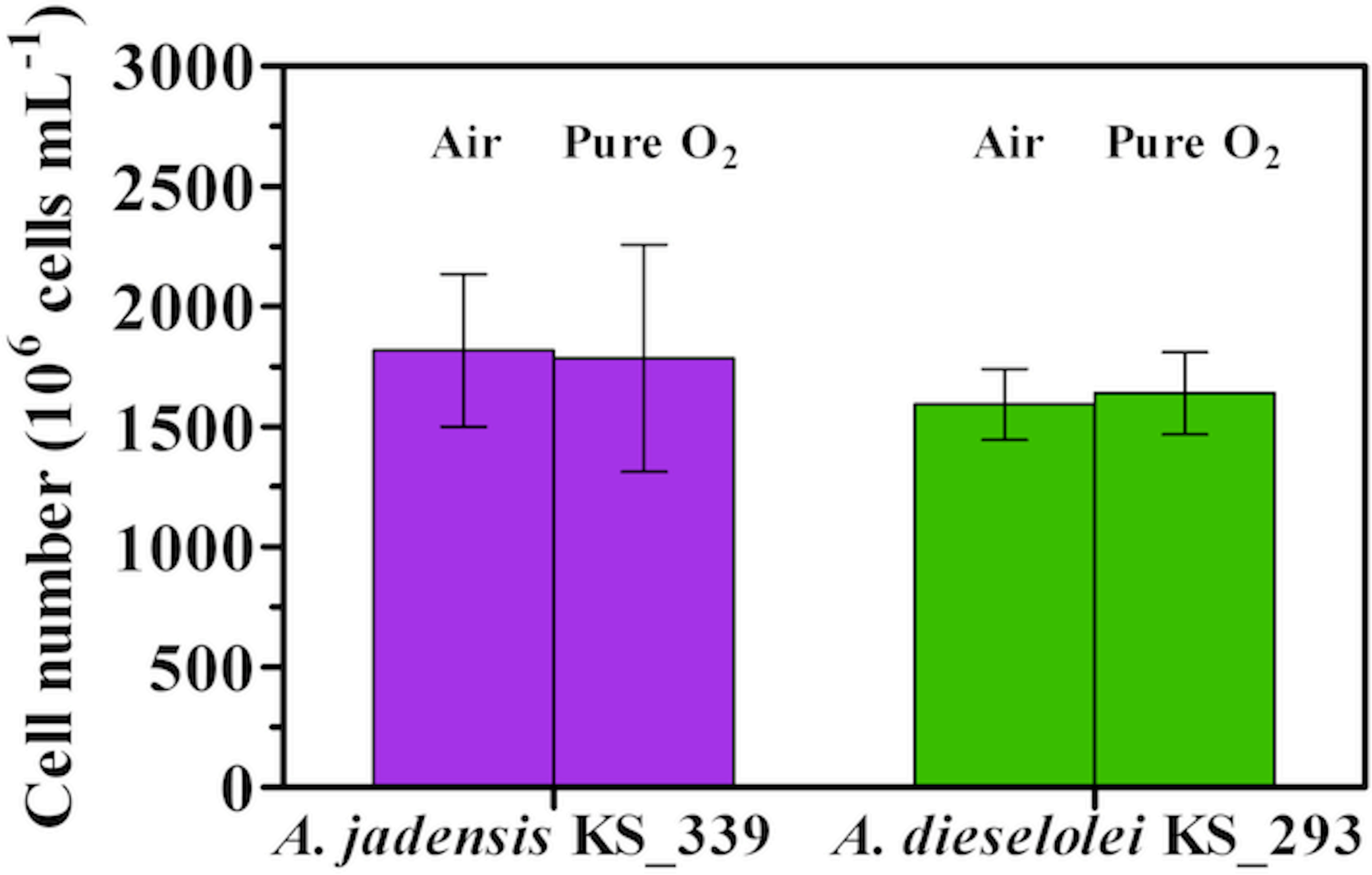

Cell number (10^6 cells mL^-1)
3000
2500
2000
1500
1000
500
0
Air
Pure O2
Air
Pure O2
A. jadensis KS_339
A. dieselolei KS_293

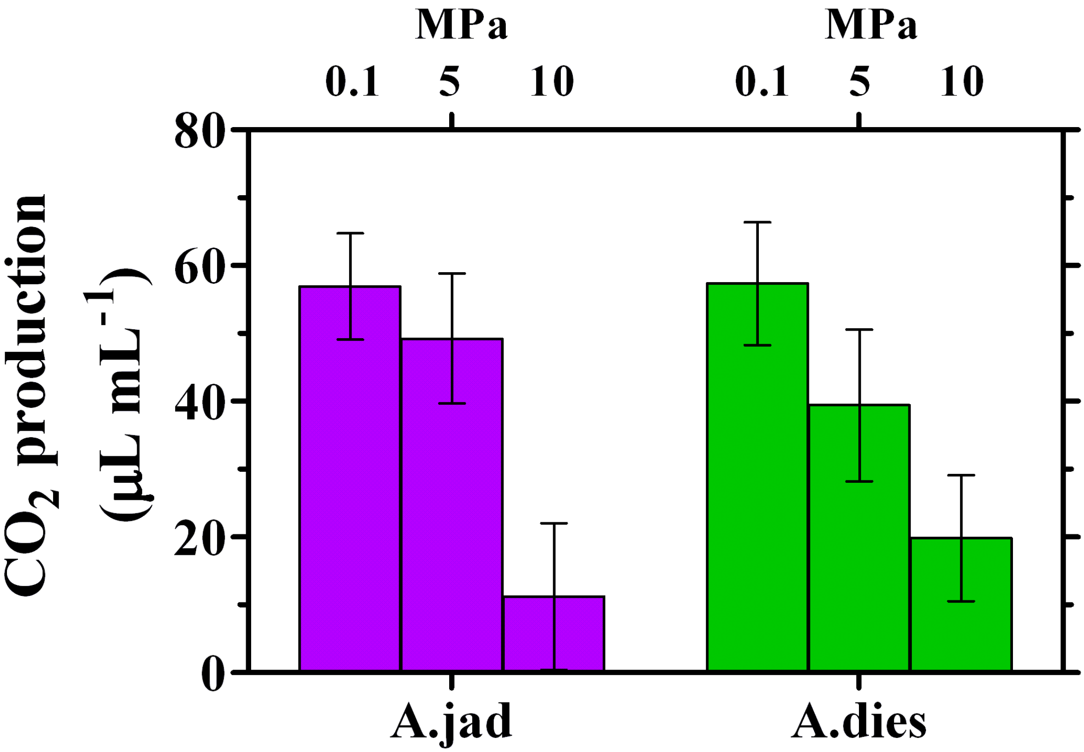

MPa
0.1
5
10
MPa
0.1
5
10
80
60
40
20
0
CO2 production (µL mL-1)
A.jad
A.dies

Table S1: Expression of some typical pressure-resistance pool of genes in *A. dieselolei* KS_293 cells under 10 and 0.1MPa

Function	Regulation	log2 FC	10MPa	0.1MPa	Cluster ID	Locus Tag	Description
Cell division							
	-	**-0.71**	64.5	105.3	1439	B5T_03495	Cell division protein FtsL
	-	**-1.59**	1.6	4.7	1370	B5T_01893	Cell division topological specificity factor
	-	**-1.84**	10.0	35.8	646	B5T_01894	Cell division inhibitor, membrane ATPase
	-	**-1.87**	6.0	21.8	449	B5T_03484	Cell division protein FtsA
	-	**-1.96**	8.5	33.0	2138	B5T_03483	Cell division protein FtsZ
	-	**-3.5**	1.0	11.8	539	B5T_03489	Cell division protein FtsW
Outer Membrane Protein	-	**-0.68**	25.1	40.2	1785	B5T_02341	OmpA family protein
	-	**-0.77**	3.9	6.6	1985	B5T_02487	OmpA family protein
	-	**-0.99**	548.4	1088.7	1217	B5T_01469	Thrombospondin type 3 repeat family, OmpA-OmpF porin, OOP family
	-	**-1.05**	9.3	19.3	927	B5T_01929	Outer membrane protein assembly complex, YaeT protein
	-	**-1.38**	18.5	48.1	965	B5T_03555	Outer membrane protein transport protein (OMPP1/FadL/TodX)
	-	**-1.74**	2.2	7.2	980	B5T_01647	Efflux transporter, outer membrane factor lipoprotein, NodT family
	-	**-1.85**	6.8	24.7	1169	B5T_01197	Outer membrane assembly lipoprotein YfgL
	-	**-2.65**	27.9	175.6	588	B5T_03706	Outer membrane assembly lipoprotein YfiO
	-	**-3.19**	0.2	1.8	1031	B5T_03240	Outer membrane protein, OMP85 family, putative
	-	**-3.24**	23.9	225.7	613	B5T_01116	OmpW family
	-	**-3.59**	0.9	10.3	531	B5T_01930	Outer membrane protein (OmpH-like)
	-	**-5.03**	0.1	2.7	1457	B5T_00661	Type I secretion outer membrane protein, TolC family
	-	**-5.2**	0.5	17.9	1416	B5T_03628	Outer membrane lipoprotein LolB
Sigma Factor	-	**-0.78**	60.4	103.6	1579	B5T_00961	RNA polymerase sigma factor
	-	**-1.11**	288.6	622.9	1243	B5T_00231	RNA polymerase sigma factor
	-	**-1.33**	6.4	16.0	1698	B5T_00495	Regulator of RNA polymerase sigma(70)
	-	**-1.73**	203.6	674.8	798	B5T_01530	RNA polymerase sigma factor
	-	**-1.79**	6.9	23.8	1798	B5T_03577	RNA polymerase sigma-54 factor
	-	**-2.95**	4.3	33.5	363	B5T_00812	RNA polymerase sigma factor, sigma-70 family
Heat/Cold Shock Proteins	=	**0.44**	85.7	63.0	412	B5T_03857	Ribosome-binding factor A; *Csp*;
	=	**0.03**	55.2	54.0	1277	B5T_03011	Hsp20/alpha crystallin family

-	**-1.88**	104.2	383.3	943	B5T_01982	Cold shock-like protein; *CspD*
-	**-1.89**	1.3	4.7	2071	B5T_03275	ATPase, histidine kinase-, DNA gyrase B-, and HSP90-like domain protein
-	**-1.98**	2.4	9.4	2179	B5T_00995	ATPase, histidine kinase-, DNA gyrase B-, and HSP90-like domain protein
-	**-2.14**	13.3	58.8	142	B5T_03709	ATPase, histidine kinase-, DNA gyrase B-, and HSP90-like domain protein
-	**-3.08**	2.1	17.3	1693	B5T_01542	ATPase, histidine kinase-, DNA gyrase B-, and HSP90-like domain protein
-	**-4.03**	0.4	6.9	382	B5T_01201	ATPase, histidine kinase-, DNA gyrase B-, and HSP90-like domain protein
-	**-4.06**	1.7	27.5	647	B5T_01484	ATPase, histidine kinase-, DNA gyrase B-, and HSP90-like domain protein
-	**-5.28**	0.4	13.8	640	B5T_00504	ATPase, histidine kinase-, DNA gyrase B-, and HSP90-like domain protein

Table S2: Total amount of O_2 transferred to the liquid phase by increased hydrostatic pressure to 10MPa. Measurements were conducted with sterile controls at 10MPa. Provided that diffusion is linear[1], at 5MPa O_2 delivered to the culture was equal to 3.09mM.

Hydrostatic pressure	O_2 in the headspace	Total head-space	Total O_2 moving to the water-phase	Total O_2 moving to the water-phase
MPa	%	mL	mL	mM
0.1	19.37 ±0.09	6.5	-	-
10	11.34 ±1.93	6.5	0.52 ±0.09	6.18 ±1.13

Table S3. Respiration capacity of *Alcanivorax* cells under 5 and 10MPa. Cell number and respiration rate values are derived from Fig. 1B and 3B

**Pressure**	***A. jadensis***	***A. dieselolei***	***A. jadensis***	***A. dieselolei***	***A. jadensis***	***A. dieselolei***
**MPa**	**Cell number (10^9 cells/mL)**		**Respiration rate (µmol $O_2/10^9$ cells)**		**mM respiration**	
5	0.86	0.79	4.1	5.7	3.6	4.5
10	0.54	0.44	10.5	8.6	5.7	3.8

References

1. Enns, T., Scholander, P.F. & Bradstreet, E.D. The effect of hydrostatic pressure on gases dissolved inwater. *J Phys Chem* **69**:389-391 (1965)
